# Developing and implementing an integrated delirium prevention system of care: a theory driven, participatory research study

**DOI:** 10.1186/1472-6963-13-341

**Published:** 2013-09-03

**Authors:** Mary Godfrey, Jane Smith, John Green, Francine Cheater, Sharon K Inouye, John B Young

**Affiliations:** 1 Academic Unit of Elderly Care and Rehabilitation, University of Leeds, Bradford Institute for Health Research, Bradford Royal Infirmary, Duckworth Lane, Bradford, West Yorkshire BD9 6RJ, UK; 2 School of Nursing Sciences, Faculty of Medicine and Health Sciences, Edith Cavell Building, University of East Anglia, Norwich Research Park, Norwich, Norfolk NR4 7TJ, UK; 3 Department of Medicine, Beth Israel Deaconess Medical Center, Harvard Medical School and Institute for Aging Research, Hebrew SeniorLife, 1200 Centre Street, Roslindale, MA 02131, USA

**Keywords:** Delirium, Prevention, Acute hospital care, Complex intervention, Implementation, Normalization process theory

## Abstract

**Background:**

Delirium is a common complication for older people in hospital. Evidence suggests that delirium incidence in hospital may be reduced by about a third through a multi-component intervention targeted at known modifiable risk factors. We describe the research design and conceptual framework underpinning it that informed the development of a novel delirium prevention system of care for acute hospital wards. Particular focus of the study was on developing an implementation process aimed at embedding practice change within routine care delivery.

**Methods:**

We adopted a participatory action research approach involving staff, volunteers, and patient and carer representatives in three northern NHS Trusts in England. We employed Normalization Process Theory to explore knowledge and ward practices on delirium and delirium prevention. We established a Development Team in each Trust comprising senior and frontline staff from selected wards, and others with a potential role or interest in delirium prevention. Data collection included facilitated workshops, relevant documents/records, qualitative one-to-one interviews and focus groups with multiple stakeholders and observation of ward practices. We used grounded theory strategies in analysing and synthesising data.

**Results:**

Awareness of delirium was variable among staff with no attention on delirium prevention at any level; delirium prevention was typically neither understood nor perceived as meaningful. The busy, chaotic and challenging ward life rhythm focused primarily on diagnostics, clinical observations and treatment. Ward practices pertinent to delirium prevention were undertaken inconsistently. Staff welcomed the possibility of volunteers being engaged in delirium prevention work, but existing systems for volunteer support were viewed as a barrier.

Our evolving conception of an integrated model of delirium prevention presented major implementation challenges flowing from minimal understanding of delirium prevention and securing engagement of volunteers alongside practice change. The resulting Prevention of Delirium (POD) Programme combines a multi-component delirium prevention and implementation process, incorporating systems and mechanisms to introduce and embed delirium prevention into routine ward practices.

**Conclusions:**

Although our substantive interest was in delirium prevention, the conceptual and methodological strategies pursued have implications for implementing and sustaining practice and service improvements more broadly.

**Study registration:**

ISRCTN65924234

## Background

Delirium (also termed acute confusion or toxic encephalopathy) is a common complication of acute illness, particularly in older people. It results in an acute disturbance of consciousness (reduced clarity of awareness of the environment), with impaired ability to focus, sustain or shift attention. It may present as a change in cognition (memory problems, disorientation and language disturbance) or perceptual disturbance (hallucinations). This develops over a short period of time (usually hours or days) and tends to fluctuate during the course of the day. Around one in five medical patients and one in three frail older people have delirium on admission [1,2] and between 12% and 18% develop delirium during their in-patient stay [3,4]. Delirium is common after surgical hip fracture repair, with 35 to 65% of patients affected [5-9].

Delirium is a distressing and frightening experience for patients and relatives [10]; and has adverse effects on recovery and functional abilities, prolonging hospital stay and increasing risks of admission to long term care and of death [7,11]. Outcomes of delirium are worse among those with severe and persistent delirium [11-13]. Adverse outcomes of mortality and new long term care admission are maintained in the medium to longer term, i.e. up to five years [14,15]. Delirium increases hospital costs and poses major difficulties for ward staff in feelings of helplessness, frustration and stress [16]; in time and resources to support those with delirium, and to manage distress caused to other patients by difficult behaviour exhibited by those suffering from it. In human, clinical, service and societal terms, delirium presents even greater costs than other adverse hospital-acquired conditions with higher policy profiles, such as falls.

There is increasing interest internationally in tackling the ‘problem’ of delirium [17,18]. However, research conducted in different health systems in North America and Europe indicate considerable disconnection between staff awareness and understanding of delirium and the weight and impact of the problems resulting from it on acute hospital wards. While current evidence concludes that delirium abatement programs have little impact on the resolution of delirium [19]; systematic reviews suggest [17,20] that delirium can be prevented among a significant proportion of those at risk of developing it. Although the precise pathways linking risk factors, vulnerabilities and susceptibility to developing delirium are not fully understood, evidence suggests that older people, those with cognitive impairment, with severe illness or who have suffered orthopaedic trauma, are particularly vulnerable in the presence of environmental, care and treatment related insults in hospital settings [21]. The most robust evidence for delirium prevention [17] relates to non-pharmacological multi-component interventions involving assessment of patients to identify and then modify risk factors associated with delirium. The conclusion drawn from this research is that about a third of incident delirium in hospitals could be prevented by this approach. Modification typically requires a complex multi-component system of care comprising education and targeted interventions directed at: optimising hydration and nutrition; reducing environmental threats; increasing orientation to time and place; improving communicative practices; supporting/encouraging mobility; and better pain and infection management. These interventions have been tested in different health systems, in diverse settings (medical, surgical and intensive care units) and employ varied modes of delivery. Service models include the Hospital Elder Life Program (HELP), [22,23] which uses a skilled interdisciplinary team assisted by trained volunteers; and pro-active geriatric consultation with targeted recommendations based on a structured protocol [24]. While HELP has been consistently effective for delirium prevention, not all prevention programmes have reported a reduction in delirium incidence [25]. Although some intervention components appear more significant than others, a high degree of protocol adherence seems critical for success [26].

Currently, a delirium prevention system of care does not exist within the UK National Health Service (NHS), although the National Institute for Health and Care Excellence (NICE) review concluded that delirium prevention should be a key priority for widespread implementation [17]. While NICE Guidelines provide a framework for developing such a system, guidelines alone are insufficient to effect change in practice and service delivery in this area [27]. This reflects the multiple levels at which change is required to successfully implement delirium prevention (organisational, professional and interdisciplinary practice), similar to complex interventions generally [28]. Further, there has been relatively low value attached to work relating to the patient care experience in acute settings, compared with medical, therapy and nursing treatment related ‘recovery’ work. This applies particularly to communicative and emotional aspects of care for older people [29] and those with dementia [30], who are particularly vulnerable to developing delirium. Finally, a major implementation hurdle is not only to introduce a delirium prevention system of care, but to embed and sustain it within routine service delivery [31].

The objective of the research programme within which the study reported here is located is to develop, test and evaluate a delirium prevention programme that would be sustainable in NHS acute care. The article presents the study development phase: a theory-driven approach to constructing a complex, integrated, multi-component intervention and implementation process aimed at embedding delirium prevention in ward practice. There are two further constituent studies planned in the research programme: a pilot to test the intervention for feasibility and acceptability; preliminary testing of the intervention in a cluster, randomised controlled feasibility study.

## Methods

### Methodology

A participatory action research approach [32] was adopted with multi-disciplinary staff teams, patient and carer representatives, voluntary service managers and volunteers to explore models of delirium prevention and delivery. This approach provided the opportunity to examine ward practice relevant to delirium and delirium prevention in the context of current clinical and experiential knowledge; to facilitate mutual learning between relevant stakeholders; and to consider strategies for implementing a delirium system of care in light of research evidence, current practice and the professional and organisational factors that shape it. This iterative, dialogic and reflexive methodology in turn informed the conceptual framework that guided data collection and analysis.

### Conceptual framework

We employed Normalization Process Theory (NPT) [33,34] as a sensitising lens through which to explore knowledge and ward practices on delirium and delirium prevention. NPT focuses on micro-social processes that affect implementation of a practice (or technique) in an organisation or clinical setting. Normalization refers to the work of individuals as they engage in activities and by which “it becomes routinely embedded in …already existing, socially patterned knowledge and practices” [34], page 541. NPT postulates four generative mechanisms that operate individually and collectively to explicate how practices (interventions) are embedded and ‘normalised’ within routine care, namely: coherence, cognitive participation, collective action and reflexive monitoring (Table 1).

**Table 1:** Normalization Process Theory: the work of implementation - four interrelated generative mechanisms

**Generative mechanism**	**Explanation**
Coherence	Individually and collectively: how the work that defines and organises a practice/intervention is understood as meaningful and invested in, in respect of the knowledge, skills, behaviours and actions required to implement it.
Cognitive participation	How the work is perceived as something worthwhile and appropriate to commit their individual time and effort [signing up] to bring about the intended outcome.
Collective action	How work practices and the division of labour through which these are carried out are modified or adapted to implement the change/intervention.
Reflexive monitoring	How participants’ individually and collectively appraise the intervention and its benefits for participants, in relation to individual and organisational goals.

After May [33]; May and Finch [34].

Whereas NPT has been developed as a tool for examining implementation processes and to enhance understanding of the implementation ‘gap’ between research and practice, we employed it to build a picture of how delirium and delirium prevention are understood as meaningful by acute ward staff and the work that staff routinely engage in on tasks relevant to prevention. The aim was to facilitate systematic consideration of the barriers and implementation strategies necessary to incorporate delirium prevention within existing acute service delivery. Specifically, we were interested in how the work of staff, individually and collectively was conducted in respect of tasks that reduced or conversely increased iatrogenic and modifiable risk factors for delirium among those who were most vulnerable. Although the value of NPT is its focus on individual and collective practices within specific settings, we were also interested in examining the wider contextual features of settings that might impact implementation [35,36]. Thus while new practices are introduced into organisations that vary in their history, culture, learning climate and readiness for change [37,38] organisational policies and practices are located in and shaped by national, political, economic and health policy contexts that in combination will affect implementation processes and outcomes [36].

### Recruitment and sampling

We recruited three hospitals in the North of England to participate; purposive selection included the availability of volunteers to test out the potential for them to contribute to the delirium prevention programme, as in the HELP model (Table 2).

**Table 2:** Details of centres recruited to the study

**Hospital**	1	2	3
**Organisation**	District general hospital	Foundation Trust	Foundation Trust
Number of beds	480	400	650
Catchment	Geographically dispersed urban and rural population	Urban, ethnically diverse population	Urban and rural population
Catchment population	200,000	350,000	300,000
Ward	Elderly Care	Elderly Care	Elderly Care
			Orthopaedic trauma
			Physical/mental health
Roles of Delirium Prevention Development Team Members	Consultant physician	Consultant physician	Consultant physician
	Senior Registrar	Staff grade physician	Directorate manager
	Staff grade physician	Senior nurse	Ward manager
	Senior nurse	Ward manager	Deputy ward sister
	Ward manager	Staff nurse	Ward clerk
	Ward clerk	HCA	Senior occupational therapist
	Senior occupational therapist	Ward housekeeper	Senior physiotherapist
	Senior physiotherapist	Senior occupational therapist	Voluntary services manager
	Occupational therapy assistant	Senior physiotherapist	Volunteer
	Physiotherapy assistant	Rehabilitation assistant	Carer representative
	Voluntary services manager	Voluntary services manager	
	Volunteer	Volunteer	
	Patient representative	Carer representative	

Although each hospital engaged volunteers, their degree of active involvement with patients on the wards and the maturity of voluntary services organisation, varied considerably between sites. Following meetings by the research team with relevant managers and clinical leads in the elderly care or orthopaedic units in each of them, agreement to participate was secured. A Delirium Prevention Development Team which included senior and frontline staff from elderly care and other wards with potential interest/role in the programme was established in each hospital (Table 2). We obtained National Health Service (NHS) research ethics committee (reference number: 10/H1302/66) and local research governance approvals for a process of consenting and engaging staff and other stakeholders in the study.

### Data collection

**Figure 1:**
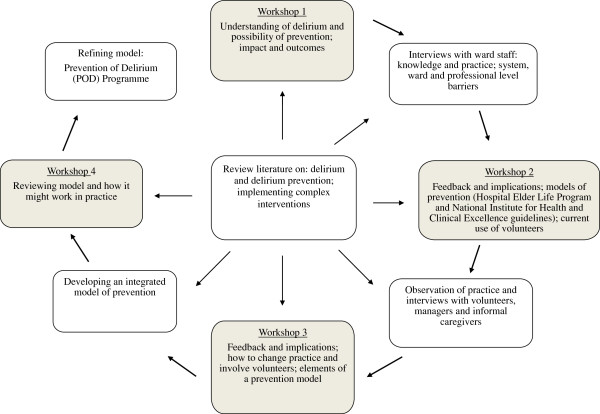
Qualitative research and development process.

Data collection by members of the research team (MG, JS, JG) who were unconnected with the clinical teams, involved multiple qualitative methods: facilitated workshops with development teams, collection of documents/records, one-to-one interviews and focus groups with multiple stakeholders, and observation of ward practices. Informed consent was obtained from participants. Four workshops with the three development teams (12 in total) were conducted over 14 months, facilitated by the researchers. Each workshop lasted around two hours. Between workshops, a detailed and nuanced picture of knowledge and practices relating to delirium prevention was garnered via qualitative interviews and observation (Figure 1).

Interviews (n=27), using a topic guide, were undertaken with 31 individuals: clinical staff (doctors, nurses and therapists in participating elderly care and trauma orthopaedic wards, in Emergency Departments (A&E) and those with a specialist/managerial role vis-a-vis older people with dementia or delirium); voluntary services managers and experienced volunteers they identified; and caregivers who had experience of caring for a relative that had developed delirium on participating wards. Fifty hours’ observations of ward practice were undertaken in the three sites at different times and on different days using ethnographic methods to expand understanding of staff routines relevant to delirium prevention in the real life, acute ward environment, supplemented by collection of relevant documents (for example, assessment forms, care plans, ward protocols, volunteer roles).

Our starting points were first the HELP model and later the NICE guidelines. The NICE Guidelines provide up-to-date evidence about the nature and scope of interventions to prevent delirium, albeit with little attention on how to embed practice and organisational change (Table 3).

**Table 3:** **National Institute for Health and Care Excellence (NICE) guidelines - risk factors and interventions to prevent delirium**^**17**^

**Risk factors**	
Age 65 years or older	
Cognitive impairment (past or present) and/or dementia	
Current hip fracture	
Severe illness (a clinical condition that is deteriorating or is at risk of deterioration)	
**Interventions to prevent delirium**	
**Clinical factor**	**Preventative intervention**
Cognitive impairment or disorientation	• Provide appropriate lighting and clear signage. A clock (consider providing a 24-hour clock in critical care) and a calendar should also be easily visible to the person at risk.
	• Reorientate the person by explaining where they are, who they are, and what your role is.
	• Introduce cognitively stimulating activities (for example, reminiscence).
	• Facilitate regular visits from family and friends.
Dehydration or constipation	• Encourage the person to drink. Consider offering subcutaneous or intravenous fluids if necessary.
	• Seek advice if necessary when managing fluid balance in people with comorbidities (for example, heart failure or chronic kidney disease).
Hypoxia	• Assess for hypoxia and optimise oxygen saturation if necessary.
Immobility or limited mobility	• Encourage the person to:
	○ mobilise soon after surgery
	○ walk (provide walking aids if needed – these should be accessible at all times).
	• Encourage all people, including those unable to walk, to carry out active range-of-motion exercises.
Infection	• Look for and treat infection.
	• Avoid unnecessary catheterisation.
	• Implement infection control procedures in line with ‘Infection control’ (NICE clinical guidance 2).
Multiple medications	• Carry out a medication review for people taking multiple drugs, taking into account both the type and number of medications.
Pain	• Assess for pain. Look for non-verbal signs of pain, particularly in people with communication difficulties.
	• Start and review appropriate pain management in any person in whom pain is identified or suspected.
Poor nutrition	• Follow the advice given on nutrition in ‘Nutrition support in adults’ (NICE clinical guidance 32).
	• If the person has dentures, ensure they fit properly.
Sensory impairment	• Resolve any reversible cause of the impairment (such as impacted ear wax).
	• Ensure working hearing and visual aids are available to and used by people who need them.
Sleep disturbance	• Avoid nursing or medical procedures during sleeping hours, if possible.
	• Schedule medication rounds to avoid disturbing sleep.
	• Reduce noise to a minimum during sleep periods.

National Institute for Health and Clinical Excellence [17]. Adapted from CG 103 Delirium: diagnosis, prevention and management. London: NICE. Available from http://guidance.nice.org.uk/CG103. Reproduced with permission.

The HELP model, initially developed in the USA from a demonstrably successful proof of concept study [23] has been widely disseminated to over 200 hospitals in the U.S., Canada, and internationally. HELP provides a skilled interdisciplinary team assisted by trained volunteers to implement standardised protocols targeted at six delirium risk factors: orientation, therapeutic activities, mobilisation, optimising vision and hearing, hydration and sleep enhancement. The core inter-disciplinary team facilitates system change and programme implementation, including daily support to volunteers (Table 4).

**Table 4:** The Hospital Elder Life Program (HELP)

**Inclusion criteria for the Hospital Elder Life Program**	
Age 70 years and older	
At least one risk factor for cognitive or functional decline. Risk factors include:	
Cognitive impairment	
Any mobility or activity of daily living impairment	
Vision impairment	
Hearing impairment	
Dehydration	
Able to communicate verbally or in writing. Nonverbal patients who can communicate in writing are included.	
**Interventions to prevent delirium**	
**Risk factor**	Preventative intervention*
Cognitive impairment	• Orientation board with names of care team members and daily schedule
	• Orienting communication
	• Cognitive stimulation activities three times daily (e.g. discussion of current events, reminiscence, word games)
Sleep deprivation	• Non-pharmacologic sleep protocol at bedtime:
	○ Warm drink (milk or herbal tea)
	○ Relaxation tapes or music
	○ Back massage
	• Unit-wide noise reduction strategies (e.g. quiet hallways)
	• Schedule readjustments to allow uninterrupted sleep (e.g. rescheduling of medications and procedures)
Immobility	• Ambulation or active range-of-motion exercises three times daily
	• Minimizing immobilizing equipment (e.g., bladder catheters, physical restraints)
Vision impairment	• Visual aids (e.g. glasses or magnifying lenses)
	• Adaptive equipment (e.g. large illuminated telephone keypads, large print books, fluorescent tape on call bell)
	• Daily reinforcement of their use
Hearing impairment	• Portable amplifying devises and special communication techniques
	• Daily reinforcement of these adaptations
	• Earwax dis-impaction as needed
Dehydration	• Early recognition of dehydration and oral volume repletion (i.e. encouragement of oral intake of fluids)
	• Feeding assistance and encouragement during meals

The “core” interventions to prevent delirium are supplemented by a number of clinical and educational “program interventions”.

* Undertaken by Elder Life staff and volunteers.

Adapted with permission from Sharon K. Inouye, M.D., MPH and the Hospital Elder Life Program, LLC.

Dissemination and embedding the programme in routine care has involved local adaptation in team composition, processes of care, procedures for patient enrolment, intervention protocols and outcome tracking [39-41]. Although the program has proven cost-effectiveness for both hospital and nursing home costs in U.S. studies [42-44] the initial costs of dedicated staff time [41] may hinder adoption and sustainability in some settings.

### Analysis

Workshop proceedings and interviews were audio-recorded, transcribed and anonymised; observational notes were written up in expanded, chronological form and all data input and stored in NVivo 9. Analysis and data synthesis was on-going and iterative. Each workshop involved feedback and discussion of emerging findings and implications from the empirical data, which in turn generated further data collection and review of evidence on implementation strategies (see Figure 1).

We used grounded theory strategies [45] such as open and focused coding and memos, constant comparison and search for negative cases, to develop categories, their constituent properties and the relationships between them. We compared and contrasted knowledge and practices relating to delirium and delirium prevention within and across wards, the professional and organisational factors that shaped them and the consequences for service delivery. The paper adheres to the RATs guidelines on qualitative research. The findings and analysis led to the development of an integrated delirium prevention programme and toolkit, iteratively elaborated and refined within Development Team workshops. The toolkit embraced intervention protocols, an implementation process and practice tools to enhance integration of delirium prevention into routine clinical practice.

## Results and discussion

In situating the task of developing a delirium prevention system of care, we describe how the work of delirium and delirium prevention was currently understood and accomplished by staff. We draw on NPT mechanisms to organise the findings and illustrate interpretive points from our fieldnotes and interview data. We then review the evolving model of delirium prevention developed iteratively through the empirical research and participatory process with development teams. Finally, we present the integrated delirium prevention programme (Prevention of Delirium (POD) Programme) including the rationale or theory of change underpinning it.

### Knowledge and awareness of delirium

While knowledge of delirium and interest in enhancing practice to prevent it was a key motivating factor for geriatricians’ involvement in the research, among other staff, awareness of delirium was more variable. Although junior doctors might be familiar with the term ‘delirium’ and knowledge based understanding was seen to have improved among registrars specialising in care of older people, there was less confidence that such knowledge was routinely translated into action to prevent delirium or manage it when it occurred. For nursing and therapy staff, delirium had not featured in their professional training. Among all staff, delirium and delirium prevention were not included as part of mandatory training or in-service education programmes. This was seen to reflect the low salience attached to delirium and delirium prevention in policy and practice such that, unlike other aspects of acute care delivery such as falls and pressure sores, there were no specific protocols relating to it in any of the sites.

Nursing, therapy and care staff generally did not use the term ‘delirium’; instead ‘confusion’ or ‘acute confusion’ were more typically employed particularly on elderly care wards: *“It's just that perhaps they don't recognise it as delirium… they don't put a label on it”* [Doctor]. Whatever the term used, among these staff, delirium was primarily understood in its manifestation as a problem for ward management and in the disruption it caused other patients. Thus, awareness (unprompted) was predominantly of hyperactive delirium that resulted in difficulties for staff from such problematic behaviour as aggression, agitation, shouting and wandering. There was acknowledgement that hypoactive delirium resulting in withdrawn, lethargic behaviour could easily be overlooked in an acute environment. Indeed, staff awareness and understanding of the experience of delirium from the perspective of patients and caregivers was prompted through presentation of the evidence by the research team, and patients’ and caregivers’ concrete accounts of specific episodes of delirium, within the workshops.

How staff perceived the nature, impact and consequences of the ‘problem’ of delirium affected how they sought to manage it. Awareness of ‘acute’ or ‘temporary’ confusion was seen to result in information seeking from family and friends to determine whether this was of longstanding duration or of recent origin:

“They might have been getting worse over a few weeks so, you really need to speak to a carer or relative; quite often we ring home care as well. We ring district nurses: ‘how are they normally, how have they been, have you noticed any change in their condition over the last few weeks’…often the consultant… if we haven't done it, will ask for us to get information from home care or whatever”. [Senior nurse]

Ascertaining that the change was recent and atypical behaviour might precipitate a search for underlying causative factors contributory to the delirium so as to identify solutions to address them, for example, sepsis (on elderly care wards) or to types of pain relief (following surgery on orthopaedic wards). The practice consequences of identifying delirium also highlighted the process whereby delirium affects treatment and lengthens in-patient stay. Therapists, for example, indicated that mobilisation might need to be delayed to allow patients the chance to recover sufficiently to engage in rehabilitation.

“That's very common [delirium with sepsis], now those patients who are… acutely ill and we feel are in that stage, we don’t always try and do anything with them in the early days because we’re aware of the fact that, say they'd come in with a UTI that sometimes just having a couple of days to recuperate means that when we intervene then they'll have a much more successful outcome… they're the sort of patients who we might discuss with the nursing staff and they'll say, leave it today, you know…[Therapist]”

Some staff used the terms ‘confusion’ and ‘acute confusion’ interchangeably. This imprecision in language use denoted a lack of clarity about the distinction between acute confusion and dementia. The practice consequences were that search for a cause might not be pursued:

*“I think a lot of the times … it's probably put down more to dementia than it is to delirium … when I guess so many people who have dementia are … more susceptible to having delirium…. And* [then for people with dementia], *I think it's probably more put down to: they're…are out of their own environment, they've had a traumatic operation, they're just more confused rather than there's perhaps another underlying issue that's causing it…”* [Therapist]

The conflation of delirium and dementia by staff was a source of heightened anxiety and perplexity among caregivers/relatives as the suddenness of the change and the strangeness of the behaviour of their relative was not understood by staff. Aggression and/or refusal to participate in treatment could be interpreted as ‘lack of engagement’, resulting in the patient being perceived as unsuitable for rehabilitation or berated by staff for ‘inappropriate’ behaviour. The following provides an illustrative example of the non-recognition of delirium and response to disruptive behaviour. Mrs Patterson’s brother-in-law was admitted to hospital “with a very high temperature and inflammation in his leg. I’m not quite sure what diagnosis was put on it”; he was also disabled following a stroke several years previously. “When he was admitted … everything ‘went …during the night he just screamed for my sister…I went down to the hospital the following morning…it was obvious to me something was wrong…he was shouting and aggressive…and demanding. The nurse said to me when I went on the ward: “oh I’m glad you’re here, I want to say this in front of you [looking at the brother-in-law] that you are a very difficult man and we don’t like the way you’re speaking to us and if you continue we will refuse to nurse you”… I said to her that he’s not like this usually… The doctor later confirmed that he had delirium”. (Note: Mrs Patterson is a pseudonym).

Generally, then, variability in how delirium was understood among different groups of staff and the lack of investment at organisational level in respect of training and education meant that delirium identification had low coherence, in NPT terms. Delirium diagnosis was primarily effected through use of observational cues – although how these were interpreted and acted upon depended on the expertise of those making the observations. Thus, management practices following on from observations reflected the skills and interests of individual professionals rather than collective staff and ward response.

### Delirium prevention

The work of delirium prevention as a meaningful set of practices posed even more difficult challenges for staff, since prevention necessitates a more complex understanding of a problem than how to manage it. Engaging in preventive action requires knowledge at different levels: about risk factors that may pre-dispose a patient to the problem, and the kinds of interventions or practices that have the potential to reduce modifiable risk. It also requires systems to identify those at risk, and the mobilisation of staff to carry out practices which contribute to risk reduction.

Given the low coherence of delirium among staff groups across sites, it is hardly surprising that delirium prevention was not perceived as meaningful, also evidenced from research in Australian [46] and Canadian [47] health systems. Even where senior staff had initiated action to increase awareness of delirium risk (e.g. posters displaying risk factors), this did not inform assessment and care practices: *“it’s not in the foreground of people’s minds”* [Geriatrician]. Interviews and observations indicated that knowledge of delirium among individual staff did not necessarily translate into specific beliefs and behaviours (cognitive participation) and the organisation of work practices geared toward prevention (collective action). The following provides an illustrative example of the non-translation of delirium knowledge into preventative action on risk.

In one elderly care ward, we observed that senior nursing staff employed the term 'delirium' in describing specific patients, demonstrated awareness about, and knowledge of both hypoactive and hyperactive delirium and sensitivity to the distress caused to patients with it. The consultant geriatrician also had a particular interest in delirium. During observation of a nursing handover meeting on this ward, it was reported that just under a fifth of current patients were characterised as having delirium. One of the delirious patients discussed was perceived as needing considerable assistance with eating and drinking; another was referred to as having 'hypoactive delirium', being 'really drowsy', 'not sufficiently alert to eat and drink', 'incontinent' and on 'IV fluids'. For the former patient, it was emphasised that all staff should be alerted to ensure support at mealtimes and to encourage drinking and eating. For the latter, it was agreed that she should be moved to a bed which was more visible from the nurses' station, although this also provoked discussion about the disorientation such a move might cause. At the same meeting, several newly admitted patients were described as having symptoms which to the observer might portend risk of developing delirium: an 89 year old patient who had suffered multiple UTI infections and been admitted following a fall; and an 80 year old patient with a urine infection and pneumonia who had suffered a heart attack and needed oxygen. The symptoms were presented without reference to, or discussion of delirium risk or any specific preventive action to be taken. This was recognised as typical practice by the Development Teams. Thus, even where there was shared understanding of delirium management, this did not facilitate noting and acting upon risk factors before delirium had occurred (cognitive participation in Normalization Process Theory).

 One exception to this general gap between knowledge and practice in delirium prevention was the development and implementation of a protocol on pain management post-surgery in respect of hip fracture patients on the trauma orthopaedic ward. This was aimed at delirium prevention. Staff remarked on how the protocol was routinely pursued with positive outcomes as a consequence, particularly in reducing severity and length of delirium episodes. The ward manager attributed success to specific features of the hip fracture patient pathway: this was direct, linear and highly protocolised with all patients diagnosed with hip fracture fast tracked from accident and emergency (A&E) department to the ward to undergo surgery within 24 hours. Insertion of the protocol into the pathway was viewed as an elaboration of existing practice rather than a major shift in how things were done. Practice change was reinforced by the perception among nursing staff that this was a relatively simple intervention with visible positive effect within a short period of time. By contrast, the patient journey into elderly care wards across the three hospitals was more protracted and diverse. Triage systems and initial investigation in A&E to determine a differential diagnosis and whether admission was warranted, might be followed by further observations within a short stay assessment facility (AAU) for up to 48 hours; which could be further protracted because of shortage of acute ward beds. The chaotic nature of A&E and AAU environments compounded by the multiple potential aetiologies of delirium in these settings were viewed as contributing to delirium risk so that the scope for preventing incident delirium on acute wards could be adversely affected by the length of the patient journey into them. Even so, delirium prevention was considered to be feasible and worthwhile within the acute ward environment, although organisational factors shaping the patient journey through the hospital also needed attention as part of a strategic approach to prevention.

### Current ward routines and practices

From interviews with staff and development team discussions, the acute care ward was reported as ‘busy’, often ‘chaotic’ and challenging: a picture reinforced by research observation. Explanatory, contributory factors offered by staff included: patient mix and the policy and organisational imperative to achieve rapid patient throughput. Policy and service emphasis on hospital admission of those who require specialist medical and nursing expertise that could not be provided in alternative settings meant that patients were very acutely ill. Similarly, it was expected that patients would move on from acute care once medical and functional needs were met to secure ‘safe’ discharge. Patient moves within wards across all sites were also common, reflecting various organisational contingencies.

The hectic nature of ward life had the consequence that routine practice was described by staff as being primarily directed at responding to what was immediately presented with priority given to diagnostic, observational and inter-disciplinary assessment and care planning. This picture was reinforced from observation. Thus, particularly for nursing and auxiliary staff, ward life was organised on the basis of a structured rhythm of time-sequenced care (washing, toileting), observations, diagnostic processes and treatment, punctuated by meals and visiting times – a pattern that was prone to disruption as a result of crisis events. Alongside this daily rhythm was the management of patient flow (admissions and discharges) and associated activities (negotiating with bed managers, discharge co-ordinators, social workers, relatives and community agencies), including record keeping.

The variability and generally poor coherence and cognitive participation of delirium prevention among staff meant that it was not a significant driver of ward care practice. However, since delirium preventative interventions relate primarily to features of care quality, it is pertinent to consider how relevant routine care practices were accomplished, including barriers and contextual factors that impacted on them.

### Nutrition, fluids and sensory aids

Although nutrition and fluid intake were viewed as components of ‘basic’ care to be undertaken by ward staff, they were primarily delivered by health care assistants (HCAs). In each site, around a third of patients required some direct help at meal-times. Others might need encouragement to eat, although this provision depended on staff availability and assumed lower priority. Similarly, tasks of washing and dressing including ensuring that patients had spectacles, dentures and hearing aids, as appropriate, were mainly undertaken by HCAs. Even so, the importance senior nursing staff attached to care tasks affected both the value attributed to them by junior nursing staff and the extent to which they pitched in to provide assistance.

### Mobilisation

Mobilisation by physiotherapy staff of patients with particular needs was limited and appeared to occur at most once daily, and was intended to be augmented with support and encouragement of ward staff. Patients who merely lacked confidence in getting up and walking on their own were reliant on nurses and HCAs to provide this. Similarly, local policies on prioritising therapy on those with the potential to resume independent living meant that for example, in one site, patients admitted from nursing homes did not receive therapy. The engagement of nurses and HCAs routinely in mobilisation work either in an enhancing or supportive role was viewed by staff as essential to sustaining mobility among patients, most of whom were of advanced age, frail and unsteady on their feet. How consistently this was done depended on such factors as the ward physical environment and other pressures.

In one site, the confined and cluttered space of the bays was a constraint on the ability of patients to move safely; and as the distance between bed and toilet was not more than a few steps, routine mobilisation by nurses and HCAs was limited. Only therapists walked patients for longer distances along the corridor where the wider space enabled freer and more confident movement. In another site, by contrast, the distance between the bed and toilet was some 10 to 20 metres. Part of the ward routine included nurses and HCAs providing direct assistance to patients and/or keeping an eye out for them as they walked from bed to toilet. It was noted over an observation period how one patient progressed from being assisted with walking to managing independently with a nurse walking behind her to walking on her own. Although here the physical environment was conducive to staff encouraging mobility, this practice was facilitated and reinforced by a care ethos which placed high value on all staff, including nurses participating in such work. This is exemplified in the following episode observed in this site but not in others. One of the nurses was with a patient as she encouraged her to stand up from being seated. As the patient made several attempts to propel herself from a sitting to a standing position, the nurse stood by continually encouraging, how to use her arms to push, moving to the edge of the chair and praising each effort, until she stood up.

### Orientation and communication

Features of the ward physical environment may act as constraints to ‘good’ care practice, for example inappropriately-placed clocks and lack of space or infection control policies precluding personal possessions.

There was variation between individual staff and professional groups within and between sites in the extent to which they conversed with patients in the course of their work. Therapists, for example, typically introduced themselves to patients they were working with, engaging them in general social and orienting conversation. The pattern was more diverse among nursing and care staff. In one site, there was a buzz of chatter in the bays as nurses and HCAs conversed socially with patients as they went about their daily routines of washing, dressing, medication rounds and mealtimes. The progress of individuals was remarked upon and patients were complemented on efforts at walking or dressing. In another site, interaction between staff and patients seemed primarily directed on the task in hand: *‘here are your tablets’; ‘do you have any pain?’*

### Cognitive stimulation and therapeutic activities

The hustle and bustle of ward life, particularly from early morning to mid-afternoon as described by staff, was in marked contrast to the silence and inactivity of patients once care needs and clinical observations were completed. We could discern two parallel but distinct ward rhythms: a staff rhythm marked by frenetic movement and continuous noise – of buzzers, telephones and the clatter of trolleys; and a patient rhythm distinguished by a paucity of conversation and little movement. Sustained or prolonged engagement of patients by staff was absent in all sites. Development teams remarked that this was neither feasible nor valued in the context of the priority attached to moving patients quickly through the system.

Overall, some practices pertinent to delirium prevention (assistance with meals for those who needed help with feeding but not for those who required encouragement) were carried out more or less consistently for some patients across all sites. Others (enabling-support to encourage and enhance mobility among patients lacking in confidence, and personally meaningful, as opposed to task-based, communication) were accomplished more consistently in some sites than others depending on local policies and priorities, the physical environment in which care was delivered and the existence of a care ethos which placed high value on social engagement and care. Yet others (spending time with patients in one-to-one conversation or engaging patients in cognitively stimulating activities) were not routinely engaged in by staff across sites; seen to reflect the current acute care environment. Collective action by ward staff in practices preventive of delirium were contingent on local policies and priorities on patient need, staffing levels, division of labour and the care culture operating. In no site were any of these explicitly linked with delirium prevention, or engaged in consistently for all patients who might exhibit delirium risk factors.

### Use of volunteers

Discussion within development teams and interviews with voluntary services managers, volunteers and ward staff revealed considerable variability in size, scope of the volunteering role, supervision arrangements, training and organisation of volunteers. One hospital had a 400-strong volunteer force since its opening some four decades previously. Here, volunteers were centrally managed under the aegis of a voluntary services manager (VSM) and deputy. The post holder was responsible for recruitment, organising training, deploying volunteers to some 30 different tasks/roles, and providing on-going support to them. In another hospital by contrast, voluntary provision was fragmented and delivered through different agencies, each focusing on discrete roles and tasks. As a consequence, there was no standardised system for recruiting, inducting, training and supporting volunteers.

Although most volunteers in all sites were primarily engaged in providing practical and orientation assistance to patients and visitors, each site had a small number of volunteers, outwith the chaplaincy service who offered one-to-one befriending with patients on the wards. These volunteers spent time conversing with patients; the purpose being to reduce isolation among those who had few visitors. They reported variable interest in what they did among ward staff ranging from positive reinforcement of the value attached to it, to indifference and hostility. Generally, staff were seen as so busy that they were unaware of volunteer input. Sustaining volunteer involvement depended on: the commitment, tenacity, skills and abilities of individual volunteers; and mutual support provided to each other through informal networks. One site had developed a successful programme for trained and supervised volunteers attached to specific wards to provide assistance and encouragement to patients who needed help with meals. A similar scheme at another had been unsuccessful, attributed to lack of attention on how to engage ward staff.

Engaging volunteers in delirium prevention tasks offered a potential resource to wards and existing direct work with patients provided the building blocks to develop it. However, the ad-hoc nature of the befriending role as typically understood by staff, the lack of clear systems for supporting volunteers, including their purposeful integration into the work of patient care, presented obstacles to releasing the potential. In NPT terms, given existing models of volunteer/ward staff engagement and practices, mechanisms for creating a common sense of purpose and value attached to the volunteer role and for establishing a division of labour that was appropriate and acceptable to both volunteers and staff, were necessary to create the conditions for involving volunteers in delirium prevention.

### Developing a model of delirium prevention

Within development teams and through iterative feedback on empirical findings, we pursued in-depth discussion on the content of a multi-component delirium prevention intervention and implementation process with particular focus at the outset on the HELP mode of delivery. With regard to the intervention, there was consensus that the NICE components and recommendations would comprise the content, since NICE extends the HELP intervention with up-to-date evidence.

One unique aspect of the HELP mode of delivery as described above is the use of trained volunteers in assisting with some of the core interventions. Development teams perceived this feature of delivery as posing major practical and conceptual difficulties, thereby challenging its feasibility in an NHS context. Practically, the level of resource required to emulate HELP was viewed as unachievable: to deliver the intervention to all patients on a typical elderly care ward would require substantial additional staffing. Conceptually, while there was considerable enthusiasm for volunteer involvement, staff considered that ward practice in respect of delirium prevention activities was central to delivering consistent, quality care such that staff needed to be actively involved in these. There was understanding among some senior staff that ward care practices, such as nutrition, fluid intake and mobilisation, were significant not only in helping to manage delirium but in having a preventive effect on its development. These practices were also viewed as pertinent to other areas of prevention which have been targeted for action at national and local policy levels to secure quality care improvements, such as falls and pressure sores. Engaging staff in the work of delirium prevention then was viewed as enhancing staff awareness of, and ascribing legitimacy to, work that has a wide spectrum preventive effect with potential to increase the quality of patient care overall.

The evolving conception of an integrated model of delirium prevention combining practice change and an enhanced role for volunteers that emerged through discussions within the Development Teams presented major implementation challenges. First, there was the paucity of knowledge and understanding of delirium prevention, particularly among nursing and care staff whose routine practices were critical in delivering preventive interventions. Second, ward staffs’ enthusiasm for involving volunteers in a more focused and direct role with patients was seen to require considerable change in the way volunteers were currently deployed. The development of such a model then required attention to both the processes and strategies for achieving practice change, and systems and mechanisms necessary to recruit, train and support volunteers to provide an enhanced and co-ordinated role within a whole ward intervention. Such changes moreover had to flow directly from knowledge and awareness of delirium prevention as worth the investment by staff individually and collectively. The Programme we developed (the Prevention of Delirium (POD) Programme) was created through the interaction of the Development Teams’ practice knowledge, current best evidence on delirium prevention [17], consideration of the findings of our empirical research and recent reviews of implementation theory and research [31,48]. The content and implementation process documented in the resultant Programme Toolkit was then further tested and refined through dialogue with the development teams.

### An integrated model of delirium prevention: POD Programme

The POD Programme is a multi-component intervention and implementation process organised in a Toolkit (Table 5).

**Table 5:** Summary of the contents of the Prevention of Delirium (POD) Programme toolkit

**Section**	**Contents**
1. Introduction	Provides the background to the programme, the theory of change underpinning it, why it is necessary, the intended objectives and the steps that need to be in place to introduce it at ward level.
2. Educational Materials	Comprises sets of slides, vignettes and case studies to be drawn upon to raise awareness of delirium and delirium prevention and create readiness for the introduction of the programme alongside involvement of ward staff.
3. Preparation for Change	Sets out a detailed implementation process, mechanisms and activities for planning the work, engaging staff, executing change and reflecting and evaluating progress and outcomes preparatory to delivery.
4. Implementation Manual	Designed to record in detail, after completion of Section 3, how each of the interventions will be implemented in routine care on the ward. This is a bespoke document, with systems and division of labour adapted to local contexts albeit addressing common functions.
5. Involving Volunteers	Specifies the detailed work involved in engaging volunteers alongside ward staff in implementing the integrated delirium prevention programme, one set of tasks that comprise part of Section 3. It is aimed at guiding the POD Action Group through those issues relating to volunteers that require discussion and decisions, for example, providing examples of volunteer role descriptions.
6. Audit and Model Tools	Provide a range of tools that may be helpful to draw upon in implementing and reviewing the outcomes of practice change.

### The POD interventions

The POD interventions comprise actions encapsulated in protocols centring on ten, targeted clinical risk factors associated with the development of delirium among vulnerable patients (Table 3). The risk factors can be organised into three distinct delirium prevention ‘bundles’ that also provide a framework for ward staff to identify what should be done and by whom, taking into account local policies and practices:

a) Action that may typically be carried out as part of medical/nursing roles (assessing and managing pain, medication management, hypoxia and infection management)

b) Action that, depending on the level of patient need, may require skilled therapy/nursing input at one end of the continuum to, at the other end, assistance provided by volunteers with appropriate competencies (for example, mobilisation, feeding)

c) Action that offers scope for volunteers to enhance care practices while stimulating practice change toward providing holistic care to patients (engaging in social and stimulating activities for which volunteers can offer a unique contribution).

### POD implementation process

POD implementation incorporates systems and mechanisms aimed at introducing, embedding and sustaining the POD interventions into routine ward care. It envisages implementation as a process and not just a single event [31]. The first step involves the mobilisation of a staff action group with the legitimacy and authority to introduce the programme and develop a plan for change that includes awareness-raising and delivering training, engaging ward staff and recruiting volunteers. The action group should comprise relevant individuals, including ward manager, matron/senior practitioner, voluntary services manager all central to co-ordinating and delivering the change although others (up or down the organisational hierarchy) may be mobilised around specific objectives and tasks.

With the action group in place, the preparation for implementation comprises staff training based on an interactive approach to foster programme coherence [49]. Educational materials present the theory of change underpinning POD interventions and facilitate consideration of current practice on identifying risk factors and preventive actions alongside practices to be implemented. These may be added to and refined depending on local need. Programme coherence is further generated through systematic review of current practices related to each of the delirium prevention interventions via staff observations and structured feedback to inform action planning. Thus, using a set of suggested, adaptable audit tools, the action group will facilitate the conduct of short periods of qualitative observation of ward practices and environment the results of which will be discussed by the ward team. This is intended both to engage the wider ward team in understanding how the intervention departs from existing practice and for securing participation in the programme of change (cognitive participation), thereby also positively impacting on ward vision and culture [36,50-52].

The next step in implementation planning involves the action group examining the interventions, one for each of the 10 clinical risk factors, with a structured approach to decision-making around allocating roles and responsibilities between staff and volunteers, informed by the audits and ward staff discussion. Implementation process activities to insert the integrated model into routine work practices comprise two sets of tasks. One set involves consideration of the appropriate role of volunteers in relation to specific delirium prevention interventions consistent with local policies. This prompts action on agreeing role descriptions, associated competencies necessary to undertake roles safely and confidently, and the appropriate training and support to do so. The other set concerns establishing and inserting into routine work practices systems and processes for the assessment and recording of clinical factors contributing to delirium risk; for communicating information and preventive tasks in respect of at-risk patients to staff and between staff and volunteers; for documenting interventions carried out by volunteers and staff; and for supervising and supporting volunteers at ward level. These activities, which have been characterised elsewhere [48] as the tasks of ‘planning, engaging, executing and reflecting and evaluating’ have the objective of enhancing ownership and commitment to the integrated model of change thereby facilitating collective action and reflexive monitoring.

Simultaneously with programme implementation planning at ward level is the recruitment of volunteers, the provision of training to support their involvement and a process of introducing them to ward staff to facilitate an integrated team approach to delivery.

The product of the planning for implementation is a bespoke POD manual with the systems, processes and division of labour in place to achieve and sustain its execution and adapted to local contexts. Even so, the principles underpinning POD and the steps in the change process to facilitate action on the intervention are standard [53]. We envisage that this is not a static document, but would be subject of regular review and change based on progress, experience and documentation of actions and outcomes [54,55].

## Conclusions

This paper and the empirical work underpinning it has focused on a key facet of complex interventions [56]: developing the components and process of implementation of a complex intervention inserted within an organisational setting (acute hospital ward) that is itself complex and dynamic.

Delirium prevention is typically little understood by staff in acute hospitals. At the same time, multi-component interventions aimed at reducing risk among those most vulnerable to developing it involve care practices that are neither consistently nor systematically carried out in routine delivery. What is not in doubt, however, are the deleterious consequences of delirium on vulnerable patients and the potential for systematic practices that address critical features of the care experience significantly to reduce its incidence among those at greatest risk. From the empirical work reported here, it is evident that systematic and purposeful engagement in practices that contribute to reducing delirium risk, while apparently straightforward, involves a complex interplay of cultural, inter-disciplinary and organisational change at ward and hospital level. At the same time, the practices that reduce delirium are those that also define care quality in acute hospital. The challenge of implementation then is at the core of securing care practice change, not only to reduce delirium but to improve care quality, particularly in respect of patients whose resilience is compromised by severe illness, cognitive impairment and frailty in advanced older age.

Building on the participatory method and empirical findings, POD offers a collaborative approach to delirium prevention involving ward staff, volunteers and patients/relatives. It is distinct from other multi-component delirium prevention programmes in several respects. First, by involving staff directly in delivering the programme, it aims to enhance a culture of care among staff on acute wards, recognising that communicating with patients and responding to their individual needs in a holistic manner are integral to promoting recovery and reducing adverse events. Second, by including volunteers alongside staff in providing that additional ‘bit of help’ (such as engaging with patients as individuals, providing cognitive and social stimulation or enhancing care through assistance with such tasks as feeding), there is the potential to increase the effectiveness of delirium prevention with an additional positive impact on the well-being of patients and the more effective use of resources. Third, although the POD Programme has a common content, it is intended to be delivered flexibly depending on pre-existing practice and local circumstances.

### Limitations and strengths

Although POD was developed through empirical work in only three hospitals, it is currently being piloted for feasibility and acceptability in three elderly care wards and two orthopaedic trauma wards in four further hospitals. Initial findings are promising, albeit some modification of the implementation process will likely be required particularly relating to systems for engaging volunteers. Following refinement, a cluster, randomised, controlled, feasibility study is proposed to explore effectiveness and cost-effectiveness of POD and to gather data to inform a future larger study.

Our study is novel in several respects. First, in employing a theory-based approach to inform understanding of the factors that shape routine practice around delirium and delirium prevention, it provides empirical support to inform understanding of the behaviours and practices that need to change to implement a preventive program. Second, it addresses a critical albeit little researched area: how to move from existing practice to develop the strategies and skills to achieve change within complex settings. Although the implementation literature offers general insights into what works to achieve change, these also need to be rooted in the concrete contexts of specific problems within their cultural, organisational and professional environments. Third, the process of developing POD as a multi-component intervention and implementation strategy through an innovatory participatory research design provides insight into a dimension of constructing complex interventions that has hitherto not received much focused attention. Thus, the methodology has potential for generalizability beyond its specific application to delirium prevention.

## Competing interests

We have no competing interests, financial or otherwise to declare.

## Authors’ contributions

MG participated in developing the research design, carrying out data collection and analysis and drafted the manuscript. JS and JG participated in the study design, carrying out data collection and analysis and helped to draft the manuscript. FC and SKI participated in the design of the study and helped to draft and revise the manuscript. JBY conceived of the study, participated in its design and co-ordination and helped to draft the manuscript. All authors read and approved the final manuscript.

## Pre-publication history

The pre-publication history for this paper can be accessed here:

http://www.biomedcentral.com/1472-6963/13/341/prepub
